# Effectiveness of Internet-Based Cognitive Behavioral Therapy for Depressive Symptoms During Pregnancy by Using Real-World Data: Retrospective Cohort Study

**DOI:** 10.2196/73512

**Published:** 2025-12-11

**Authors:** Asuka Takae, Natsu Sasaki, Kotaro Imamura, Daisuke Nishi

**Affiliations:** 1 Department of Mental Health Graduate School of Medicine The University of Tokyo Bunkyo-ku, Tokyo Japan; 2 Department of Digital Mental Health Graduate School of Medicine The University of Tokyo Bunkyo-ku, Tokyo Japan

**Keywords:** antenatal depressive symptom, prevention, internet-based cognitive behavioral therapy, real-world data, digital mental health, mobile health

## Abstract

**Background:**

Approximately 1 out of 5 pregnant women develops depression. Internet-based cognitive behavioral therapy (iCBT) is an effective way to treat not only depression but also mild depressive symptoms or subthreshold depression. While numerous iCBT programs have been developed and tested through randomized controlled trials for various mental health conditions and specific populations, research on their effectiveness and application in the real world remains limited.

**Objective:**

This study aimed to examine the effectiveness of a previously developed iCBT program implemented in an existing app for improving depressive symptoms among pregnant women in a real-world setting.

**Methods:**

The previously developed iCBT program for preventing perinatal depression was already implemented in an existing app called Luna Luna Baby by MTI Ltd. The app aims to provide information to pregnant women about pregnancy and babies, and potential users can download it from the Japanese version of the Apple App Store or Google Play Store without any fee. The program does not require any additional fees. The log data stored on the app identified iCBT program users and nonusers, allowing us to conduct this retrospective cohort study. Data from September 2022 to September 2024 were extracted from the app after anonymous processing. The primary outcome was the score on the self-reported Edinburgh Postnatal Depression Scale (EPDS), which participants answer by themselves on the app. The exposure group was defined as completers of all 6 modules of the iCBT program. The nonexposure group was defined as users who did not use any module of the program and matched the baseline characteristics of the exposure group. The change in EPDS score before and after using the program was compared using effect sizes, and repeated 2-way ANOVA was conducted to test the difference between the exposure and nonexposure groups.

**Results:**

Data from 119 women who completed the iCBT program and 448 pair-matched controls were selected. The average EPDS scores at baseline were 7.24 (SD 5.30) in the exposure group and 7.25 (SD 5.18) in the nonexposure group. After using the iCBT program, the group mean EPDS scores changed by −0.69 (SD 4.92) and +0.99 (SD 5.56) over time in the exposure and nonexposure groups, respectively (Cohen *d*=0.31, 95% CI 0.11-0.51). The repeated 2-way ANOVA showed statistical significance in the interaction terms between the groups and the measurement time points (*P*=.04).

**Conclusions:**

The previously developed iCBT program showed a significant effect with a modest effect size on decreasing depressive symptoms among pregnant women in a real-world setting. Future research should attempt to minimize dropouts and increase participation in the program.

## Introduction

Depression is common among pregnant women, with a global prevalence of antenatal depression estimated at 20.7% [1]. In Japan, prevalence rates are 14% during midpregnancy and 16.3% in late pregnancy [1,2], suicide is the most frequent cause of perinatal death, and depression is the major risk factor for suicide [3]. Depressive symptoms during pregnancy affect not only pregnant women themselves but also fetuses and babies and can lead to adverse outcomes for them, such as impaired physical growth, preterm birth, low birth weight, decreased reactivity, changes in temperament, and behavioral disorders [4-6]. Therefore, it is essential to work on preventing depression among pregnant women.

Cognitive behavioral therapy is a way to prevent depression during pregnancy [7]. Internet-based cognitive behavioral therapy (iCBT) has recently increased accessibility to this therapy modality and convenience. Although iCBT was initially applied for treating depression, a recent study reported that iCBT was also effective for those who have mild depressive symptoms or subthreshold depression [8,9]. An iCBT program for preventing depression among pregnant women on a smartphone app has already been developed and investigated for its effectiveness in a randomized controlled trial (RCT) in Japan [10,11]. This RCT did not find an overall effect of the program in preventing perinatal depression; however, post hoc analyses suggest potential effectiveness among individuals with mild psychological distress. The program is now available as a free service on a popular Japanese app for anyone to use [12].

RCTs are conducted in a strictly controlled setting, which may be inconsistent with the real world. Significant differences have been reported between study populations from RCTs and study populations from real-world data, even using the same sampling criteria [13]. Engagement with mobile mental health apps is also lower in the real-world setting than in the clinical trial setting [14,15]. Therefore, it is necessary to confirm whether the developed iCBT program is effective not only in study settings but also in real-world settings to ensure its external validity [16-18]. Even though several studies have evaluated the actual use of digital interventions and identified their effectiveness for the treatment of depression and anxiety [19-23], we found no such efforts for iCBT programs for prevention or among pregnant women.

The objective of this observational retrospective cohort study was to investigate the effectiveness of iCBT use among pregnant women in improving depressive symptoms in a real-world setting.

## Methods

### Study Design and Data Setting

This observational study was conducted based on a previous RCT [10]. The users evaluated the iCBT program highly in terms of acceptability, appropriateness, fidelity, and satisfaction [24]. After the study, this developed iCBT program was implemented in the real world. The iCBT program was released in an existing app for perinatal women called Luna Luna Baby by MTI Ltd as a free standard feature in September 2022 [12]. Luna Luna Baby is a smartphone app that aims to provide pregnant women with information about pregnancy and babies. The app can be downloaded from the Japanese version of the Apple App Store or Google Play Store without any fee, and it does not require any additional fees to use the program on the app. A self-assessment service based on the Edinburgh Postnatal Depression Scale (EPDS) questionnaire was also implemented on the app at the same time, allowing pregnant women who use this app to check their current mental status whenever they want. The app routinely logs the results of users’ self-checked EPDS scores. These data include not only the EPDS score and its measurement date but also basic information about the user at the time of EPDS measurement, such as age, weeks of pregnancy, and the number of times the user read the iCBT modules. This study used the sample from September 2022 to September 2024 extracted from these EPDS service log data after anonymous processing. There is no linkage across other databases. Given that user access typically decreases with increasing depth in a hierarchical app structure, we collected information on the number of visits to pages at each hierarchical level. We also collected information regarding any modifications made to the structure over the observational period. This allowed us to analyze user navigation patterns during this period. These data were all provided by MTI Ltd, the app developer.

### Participants and Measurements

#### Participant Selection

**Figure 1:**
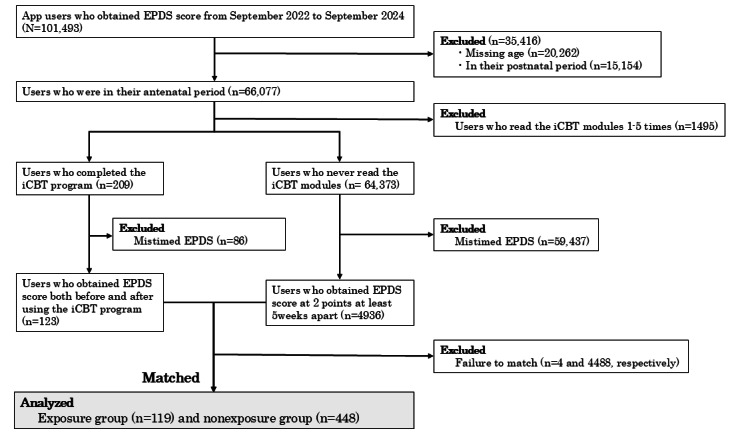
Participant selection flowchart. EPDS: Edinburgh Postnatal Depression Scale; iCBT: internet-based cognitive behavioral therapy.

Pregnant women who had downloaded and used the Luna Luna Baby app from September 2022 to September 2024 were possible participants (N=101,493). Among them, those who were in their antenatal period were included (66,077/101,493, 65.1%). Those who were in their postnatal period or those with missing age data were excluded. The exposure group was defined as those who completed the iCBT program (209/66,077, 0.31%). The nonexposure group was defined as those who never read the iCBT modules (64,373/66,077, 97.42%). To compare changes in depressive symptoms, participants were limited to those who had EPDS scores from 2 time points, before and after exposure to the iCBT program (123/209, 58.9%). For the nonexposure group, participants were limited to those who had EPDS scores from 2 time points at least 5 weeks apart based on the estimated iCBT program duration (4936/64,373, 7.66%). The details of the selection are shown in Figure 1.

#### Exposure: iCBT Program Use

##### Exposure Group

The iCBT program consists of a total of 6 modules, each including psychoeducation, case formulation based on a cognitive behavioral model, behavioral activation, self-compassion, mindfulness, and problem-solving. All modules were based on the topics that many women tend to be concerned about during pregnancy. The details of the module contents have been explained elsewhere [11]. The iCBT program within the app becomes accessible only after 16 weeks of pregnancy, after which users may access one module per week—each requiring approximately 5 minutes for completion—at a time convenient to the user. Although the program can be completed within 5 weeks at minimum, it can be used at any time as long as the users have the app downloaded. The program’s progress is managed by clicking the “Read” button at the bottom of each module page, and the next module is opened 1 week after clicking the button on the previous module page. The number of modules read is recorded when the EPDS is measured, which enables us to identify the timing of program use from EPDS log data.

##### Nonexposure Group

A previous study has demonstrated that baseline factors such as maternal age and stress levels influence the trajectories of depressive symptoms from pregnancy to the postpartum period [25]. In particular, the severity of depressive symptoms at baseline has been shown to affect the efficacy of interventions [10,26]. Therefore, to ensure comparability between the exposure and nonexposure groups, participants in the nonexposure group were selected using matching based on the following baseline variables with the exposure group: age, weeks of pregnancy, and total EPDS score. The caliper widths were set at –2 to +2 for age, –1 to +1 for weeks of pregnancy, and –1 to +1 for total EPDS score following previous research [27-29].

The total EPDS score was added in this study as a matching variable because it was suggested that baseline severity of depressive symptoms affects intervention effects [10,26]. We randomly selected up to 4 controls per iCBT program completer without replacement, such that any individual selected as a control could not be chosen again for another case within the study [30,31].

#### Outcome: Antenatal Depressive Symptoms

To evaluate depressive symptoms during pregnancy, we used the Japanese version of the EPDS [32,33]. This scale consists of 10 questions about perinatal-specific depressive symptoms measured on a 4-point scale ranging from 0 to 3, with a total score ranging from 0 to 30. A higher score indicates more severe depressive symptoms. A cutoff of 12 or 13 is considered appropriate for antenatal depression [34]. In addition, a cutoff of 8 or 9 is often used clinically in Japan to screen for perinatal depression [32], so this cutoff was also considered. Although participants could answer the questionnaire only at given time points (eg, 16 to 20 weeks of pregnancy and 32 weeks of pregnancy) in the previous RCT [10], after the launching the service in public, app users can answer the EPDS whenever they wanted to check their mental status. The app displays advice to the users depending on the score obtained. When the score is 0 to 8, the advice is as follows: “There seems to be no problem with your mental status. Let’s continue with the self-check of your mental status.” When the score is 9 to 12, the advice is as follows: “You may not be feeling well. We recommend that you take extra care of yourself by getting enough sleep, taking time for yourself, and doing activities that will refresh you. Please also use our Pre-mother School modules.” When the score is 13 or higher, the advice is as follows: “It is possible that your mental status is not at its best. We recommend that you take better care of yourself than usual and consult with a trusted family member, friend, supervisor, or colleague. If you continue to be in the wrong mood, please consider consulting your obstetrician-gynecologist or public consultation service.” These messages appear automatically, and there is no tracking of what actions participants take afterward. The app also encourages users to answer the EPDS using push notifications. All users regardless of exposure to iCBT receive a notification to answer the EPDS when they access the “Mental Care” section with the following message: “Do a self-check once every two weeks.” For users who have previously completed an EPDS assessment, a follow-up notification is sent 2 weeks after their last measurement. These notifications are not linked to iCBT program use status and are delivered uniformly across users.

#### Process Evaluation

**Figure 2:**
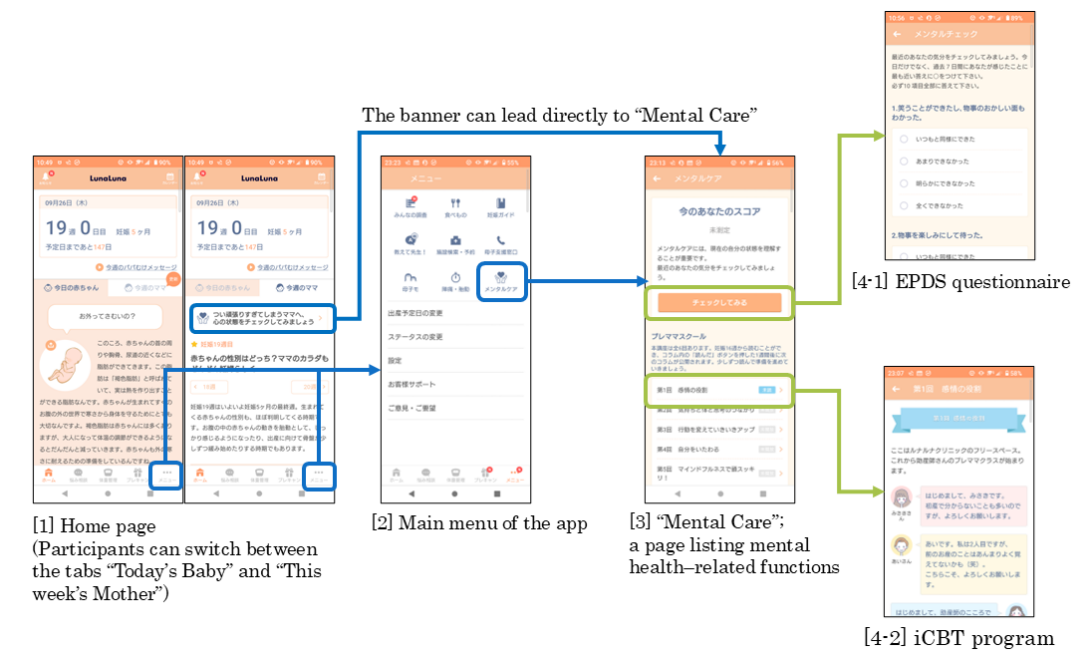
Screenshots of the Luna Luna Baby app. EPDS: Edinburgh Postnatal Depression Scale; iCBT: internet-based cognitive behavioral therapy.

We reported the reach and completion rate of the iCBT program among users who downloaded the app. After launching the service to the public, both the iCBT program and the EPDS questionnaire were stored on a higher-level page named “Mental Care.” The “Mental Care” page lists mental health–related functions, 2-levels-lower from the top page (Figure 2). In February 2023, the specifications were updated to include a banner that enables users to jump from the home page to the “Mental Care” page with a single click. The banner shows the following message: “For mothers who try too hard, let’s check your mental state.” Under these specifications, “reach” was defined as the proportion of those who visited the “Mental Care” page, iCBT program page, and EPDS page at least once each. The completion rate was defined as the proportion of those who completed all 6 modules.

#### Covariates

Age and weeks of pregnancy at the time of the baseline EPDS score were measured and used as basic information. For service use information, the observational period was defined as the period between the baseline and follow-up time points when EPDS scores were measured because the log data did not include date information about iCBT program use. The number of additional EPDS measurements at time points within the observational period other than baseline and follow-up was also calculated.

### Statistical Analysis

First, the baseline characteristics of the exposure and nonexposure groups were compared using standardized differences. Service use information was also compared between groups using 2-tailed *t* tests. We calculated changes in EPDS scores after program use, 95% CIs, and Cohen *d* effect sizes to assess the association between the iCBT program and depressive symptoms. Repeated-measure ANOVA was also conducted to identify the statistical significance of interaction terms between the groups and the measurement time points. Regarding the process evaluation, reach and completion rate were calculated using information on the number of users visiting the “Mental Care” page, iCBT program page, and EPDS questionnaire page per month. All analyses were conducted using R (version 4.4.1; R Foundation for Statistical Computing), and *P*<.05 was set as statistically significant.

### Ethical Considerations

This study was approved by the Research Ethics Committee of the Graduate School of Medicine and the Faculty of Medicine of the University of Tokyo (approval 2019150NI). Informed consent to collect data from the app was obtained from users who had an EPDS score after informing them that the data would be used for research purposes. The data from the app were anonymously processed. There was no compensation provided to participants for this study as we tracked natural use of the app.

## Results

### Participant Characteristics

Of the 101,493 women (173,712 records) who answered the EPDS on the app from September 2022 to September 2024, a total of 209 (0.2%; 1675 records) completed the 6 iCBT modules, and 64,373 (63.42%; 101,251 records) never read any of the iCBT modules. After excluding those without EPDS measurements before and after using the iCBT program or those who were excluded from the matching process either because they did not fall within the specified caliper range for one or more matching variables or because there were more than 4 eligible control candidates for a single exposed participant, 119 women who completed the iCBT program and 448 pair-matched controls were identified.

All variables showed standardized differences of <0.1, which suggests that baseline characteristics were balanced between the exposure and nonexposure groups after matching (Table 1). The mean age of the participants was 33.31 (SD 4.12) years and 33.04 (SD 3.85) years in the exposure and nonexposure groups, respectively. The mean weeks of pregnancy at baseline were 18.00 (SD 5.01) and 17.63 (SD 4.85) in the exposure and nonexposure groups, respectively. The mean EPDS scores were 7.24 (SD 5.30) and 7.25 (SD 5.18) in the exposure and nonexposure groups, respectively. Demographic data of the full sample (N=101,493 women; 173,712 records) are shown in Multimedia Appendix 1, with a mean EPDS score of 8.33 (SD 5.71) to 8.53 (SD 5.88). The prevalence of antenatal depressive symptoms above the EPDS cutoff of 12 or 13 was 15.9% (19/119) and 17.4% (78/448) of the participants in the exposure and nonexposure groups, respectively. The mean observational period was 11.67 (SD 5.80) weeks in the exposure group and 13.61 (SD 6.13) weeks in the nonexposure group (*P*=.002). The mean number of additional EPDS measurements during the observational period was 3.17 (SD 1.70) in the exposure group and 0.51 (SD 0.95) in the nonexposure group (*P*<.001).

**Table 1:** Baseline participant characteristics after matching.

Variable	Exposure group (n=119)	Nonexposure group (n=448)	Standardized difference
Age (y), mean (SD)	33.31 (4.12)	33.04 (3.85)	0.068
Pregnancy duration (wk), mean (SD)	18.00 (5.01)	17.63 (4.85)	0.075
EPDS^a^ score (0-30), mean (SD)	7.24 (5.30)	7.25 (5.18)	0.002
EPDS score of ≥9, n (%)	40 (33.6)	147 (32.8)	0.017
EPDS score of ≥13, n (%)	19 (16.0)	78 (17.4)	0.039

^a^EPDS: Edinburgh Postnatal Depression Scale.

### Antenatal Depressive Symptoms Before and After Using the iCBT Program

**Figure 3:**
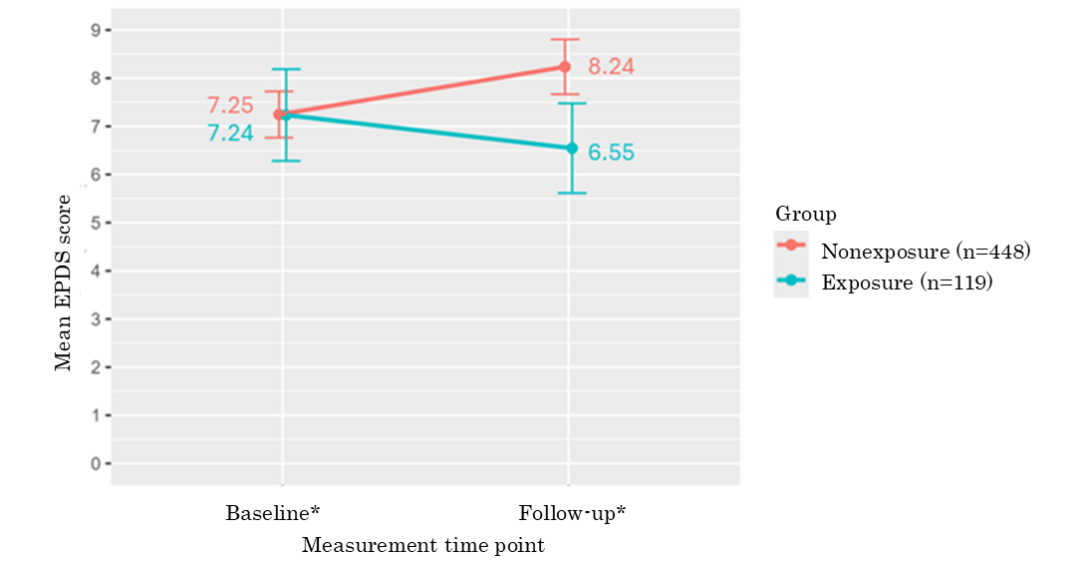
Changes in the EPDS score after iCBT program use. EPDS: Edinburgh Postnatal Depression Scale; iCBT: internet-based cognitive behavioral therapy. *The mean observational period between baseline and follow-up was 11.67 (SD 5.80) weeks in the exposure group and 13.61 (SD 6.13) weeks in the nonexposure group.

Table 2 and Figure 3 show the EPDS scores and the differences before and after using the program. The average EPDS score at baseline was 7.24 (SD 5.30) in the exposure group and 7.25 (SD 5.18) in the nonexposure group. Compared to before using the program, the EPDS scores changed by −0.69 and +0.99 in the exposure and nonexposure groups, respectively (Cohen *d*=0.31, 95% CI 0.11-0.51). The repeated-measure ANOVA showed statistical significance in interaction terms between the groups, and the measurement time points (*P*=.04).

**Table 2:** Changes in the depressive symptoms after the internet-based cognitive behavioral therapy intervention.

Variable	Exposure group (n=119), mean (SD; 95% CI)	Nonexposure group (n=448), mean (SD; 95% CI)	Effect size (Cohen *d*)
EPDS^a^ score at baseline (0-30)	7.24 (5.30; 6.27 to 8.20)	7.25 (5.18; 6.76 to 7.73)	—^b^
EPDS score at follow-up (0-30)	6.55 (5.19; 5.60 to 7.49)	8.24 (6.16; 7.66 to 8.81)	—
Change in EPDS score	−0.69 (4.92; –1.58 to 0.20)	0.99 (5.56; 0.47 to 1.51)	0.31

^a^EPDS: Edinburgh Postnatal Depression Scale.

^b^Not applicable.

### Process Evaluation

In terms of reach, 5.4% of app users who visited the “Mental Care” page. Of these, 26.5% visited the iCBT program page, and 67.5% visited the EPDS page. Regarding completion rate, 2.6% of those who visited the iCBT program page at least once completed all 6 modules. When the population was limited to those who visited the program page and clicked the “Read” button at least once, 32.3% completed the 6 modules.

## Discussion

### Principal Findings

This study examined the effectiveness of the iCBT program for pregnant women in preventing depressive symptoms during pregnancy in a real-world setting. This study found a statistically significant trend in the reduction in depressive symptoms after using the iCBT program in the exposure group compared with the nonexposure group. The effect size of the program for reducing depressive symptoms was 0.31 (95% CI 0.11-0.51). This is similar to a previous meta-analysis that reported an effect size of 0.28 for smartphone app interventions aimed at treating depressive symptoms [18], suggesting that this iCBT program also had a mild effect on preventing depressive symptoms among pregnant women.

This study showed that the average EPDS score at baseline was 7.24 (SD 5.30) in the exposure group and 7.25 (SD 5.18) in the nonexposure group, which indicated that the participants in this study had mild depressive symptoms under the EPDS cutoff score of 9. The finding that this iCBT program was effective among those with mild depressive symptoms was consistent with those of a previous meta-analysis and RCT [8,9], which showed that unguided iCBT was slightly less, but also similarly significantly effective for those who have mild depressive symptoms or subthreshold depression compared to guided iCBT. This unguided iCBT program for pregnant women may also play a role in preventing depression symptoms from exceeding the cutoff point. The baseline EPDS scores in this study were higher than the baseline EPDS scores reported in a previous RCT that evaluated the effectiveness of this iCBT program [10]. In that study, individuals with mild psychological distress at baseline had EPDS scores of 5.66 in the intervention group and 5.45 in the control group. The differences might be due to the participants’ characteristics. The log data of this study included only those who voluntarily obtained EPDS scores at least once. A previous study showed that women with antenatal depression symptoms at baseline were more likely to use the screening features of pregnancy-related apps and spend more time using apps [35]. It is possible that the potential participants in this study had mild mental health issues and greater motivation to learn about mental health. When selecting final participants from the full sample to investigate the change in depressive symptoms at 2 time points, it is possible that some outliers who had more severe distress were dropped. It should be noted that this study was unable to observe the effects on those with particularly severe symptoms among those who obtained EPDS scores. Future studies are needed to detect the effectiveness of this program for those who have more severe distress.

The number of additional EPDS measurements during the observational period was higher in the exposure group than in the nonexposure group. It has been pointed out that repeated scale measurements themselves may have a transformative effect on cognition and behavior [36]. It has also been reported that repeated measurements of self-reported questionnaires have little impact that might raise serious concerns [37]. A study examining the effects of stress screening in the workplace and the efforts to improve the work environment found that stress screening alone did not improve workers’ psychological distress. Significant improvements were observed only when both stress screening and efforts to improve the work environment were implemented together [38]. Considering these studies, it may be reasonable to assume that the EPDS measurement itself in this study had little effect on depressive symptoms.

The average proportion of visitors to the “Mental Care” page among app users was 5.4%. This was similar to the results of a previous study, which reported a 4% open rate by daily active users of popular mental health apps in the real world [15]. Even though the iCBT program and the EPDS questionnaire were stored on the same “Mental Care” page, the visit rates were more than double, at 26.5% and 67.5%, respectively, than visits to the “Mental Care” page. This may suggest that many users who visited the “Mental Care” page were aiming to self-check their mental health using the EPDS questionnaire, not to use the iCBT program. Furthermore, the completion rate of the iCBT program was 2.6%. This result is consistent with previous reports that a digital mental health intervention could only attract participants to the first module and many people dropped out before reaching the final module [15,19,39]. However, when the population was limited to those who clicked the “Read” button at the bottom of the module pages at least once, which indicates at least some engagement, the completion rate increased to 32.3%. This was close to the completion rate of 37.2% in the previous RCT [10]. This suggests that the program may be feasible for a subset of app users in real-world settings. It is also possible that the requirement to click the “Read” button after reading the module contents unintentionally hindered program progress. Further study will be needed to reveal a way to increase participation in the program.

### Limitations

This study has several limitations. First, the population selected for effectiveness evaluation did not represent all the app users as only 5.4% of app users visited the “Mental Care” page and 26.5% of them visited the iCBT program page. Furthermore, only 2.6% completed all 6 modules among those who visited the iCBT program page. To evaluate the effectiveness of the iCBT program with more app users, including stratified analysis by the number of iCBT modules completed, it may be necessary to improve the program’s delivery environment. Although the number of participants was small, we were still able to evaluate the effectiveness of the iCBT program on those who completed it in a real-world environment. The final participants of this study were also limited compared to the app users who obtained EPDS score because of the study design, which compared the change in depressive symptoms between 2 time points among similar populations. While estimating the average treatment effect in the broader population is an important future direction, establishing the effectiveness of the program in engaged users is a necessary and informative first step. Second, there was a difference in the baseline and follow-up time points between individuals because participants were able to complete the EPDS at any time after accessing the app, resulting in variability in when these measurements were taken. The observational period was 2 weeks longer in the nonexposure group. As depressive symptoms are reported to remain stable between the middle and late stages of pregnancy [2], it is likely that the 2-week extension in the follow-up period for the control group did not substantially affect the outcomes. However, considering the relatively short time frame of this study, the possibility that this extension influenced the results to some extent cannot be entirely excluded. Third, there is a possibility of unmeasured confounding. The log data used in this study only included EPDS scores and related information; no socioeconomic factors, such as economic status, educational level, and employment status, could be considered. Previous studies have identified higher educational level and employment status as predictors of seeking mental health support through the internet [40]. It has also been suggested that, when digital mental health interventions are offered at no cost, there was not a reduction in willingness to use these interventions among individuals from socioeconomically disadvantaged groups, potentially contributing to a reduction in health disparities [41]. In this study, the iCBT program was available within the app completely free of charge, which may minimize the economic barrier. However, the engagement with mental health features or its effect may still be influenced by differences in health awareness associated with educational and employment backgrounds. Further research is warranted to clarify the impact of socioeconomic factors on effectiveness. Fourth, although the findings were statistically significant, their clinical relevance remains open to further consideration. Finally, despite this iCBT program targeting perinatal depression prevention, only the effect on antenatal depressive symptoms could be evaluated because few app users continued to use the EPDS measurement service after delivery.

### Conclusions

An iCBT program targeting the prevention of perinatal depression was significantly effective in decreasing depressive symptoms among pregnant women who downloaded the app and engaged with it in a real-world setting. Future studies should explore efforts to reduce dropouts and increase participation in the program to help more pregnant women reduce their depressive symptoms.

## Data Availability

The datasets generated or analyzed during this study are not publicly available because data sharing was not approved by the institutional review board, but are available from the corresponding author on reasonable request.

## Appendix

Multimedia Appendix 1 Demographic data of the full sample.

## Abbreviations

EPDS: Edinburgh Postnatal Depression Scale
iCBT: internet-based cognitive behavioral therapy
RCT: randomized controlled trial
