# Supplementary material for: Effectiveness of Internet-Based Cognitive Behavioral Therapy for Depressive Symptoms During Pregnancy by Using Real-World Data: Retrospective Cohort Study
Source: JMIR Mhealth Uhealth. 2025 Dec 11;13:e73512. doi: 10.2196/73512 (PMC12741654; doi:10.2196/73512)
Supplement: Multimedia Appendix 1 [file mhealth_v13i1e73512_app1.docx]

[Supplementary table 1] Demographic data of the full sample

| Variables | The full sample | | | |
| --- | --- | --- | --- | --- |
|  | unique participants ^a^ | | record-based participants ^b^ | |
|  | N=101,493 | | N=173,712 | |
|  | Mean (SD); n(%) | | Mean (SD); n(%) | |
| Age(years) | 32.12 | 11.10 | 32.25 | 10.04 |
| Weeks of pregnancy(weeks) | 14.12 | 9.80 | 16.22 | 10.47 |
| EPDS score | 8.33 | 5.71 | 8.53 | 5.88 |
| EPDS≧9 | 43,141 | 42.5 | 76,246 | 43.9 |
| EPDS≧13 | 22289 | 22.0 | 40,287 | 23.2 |
| SD: standard deviation, EPDS: Edinburgh Postnatal Depression Scale | | | | |
| ^a^ mean using the median value for one person when the person has multiple record. | | | | |
| ^b^ mean using all record including multiple measurement for one person. | | | | |

a
